# Supplementary material for: Validation of the Japanese Version of the Yale Food Addiction Scale 2.0 (J-YFAS 2.0)
Source: Nutrients. 2019 Mar 22;11(3):687. doi: 10.3390/nu11030687 (PMC6471687; doi:10.3390/nu11030687)
Supplement: Supplementary file 1 [file nutrients-11-00687-s001.pdf]

Table S1: The Japanese version of the Yale Food Addiction Scale 2.0 (J-YFAS 2.0) (written in Japanese)

イエール食物依存調査票バージョン 2.0 日本語版 (J-YFAS 2.0)

この調査は、あなたの過去 1 年間の食習慣を尋ねています。

以下に示したような特定の食品をたくさん食べてしまうのをやめるのが困難な人がいます。

- 甘い物（アイスクリーム、チョコレート、ドーナツ、クッキー、ケーキ、キャンディ、和菓子等）
- 炭水化物（パン、パスタ、米等）
- 塩味のスナック（ポテトチップス、プレッツェル、クラッカー、インスタントラーメン等）
- 脂肪分の多い食品（ステーキ、ベーコン、ハンバーガー、ピザ、フライドポテト等）
- 砂糖入り清涼飲料水（炭酸飲料水、果汁入り清涼飲料水、スポーツドリンク、栄養ドリンク等）

以下の質問で「特定の食品」という用語が出てきた場合は、上に例示した食品・飲料に似た食品・飲料や、過去 1 年間に問題を生じさせていた食品・飲料を考えて答えてください。

過去 12 か月の間に	全くない	月1回未満	月1回	月2～3回	週1回	週2～3回	週4～6回	毎日
1. 特定の食品を食べ始めると、考えていたよりもずっと多く食べていた								
2. もはや空腹ではないのに特定の食品を食べ続けていた								
3. 身体的な不調を感じるまで食べていた								
4. 特定の食品を食べるのを減らすようすごく気にしていたが、やっぱり食べてしまった								
5. 食べすぎたせいで、だるさや疲れをとても感じた								
6. 1 日の中でとても長い時間を特定の食品を食べることに費やした								

7. 特定の食品がない時、それを入手するために出かけた（例：他に食べる物が家にあるのに、その食品を買うために店に行った）								
8. 特定の食品をとても頻繁に、あるいはとてもたくさん食べるので、他の大事なこと（例：仕事、家族や友人と共に過ごす等）をするのをやめてしまった								
9. 私がどれだけたくさん食べるかが原因で、家族や友人との間で問題が起こった								
10. そこで食べ過ぎるかもしれないという心配のため、仕事や学校、その他の社会的活動を避けた								
11. 特定の食品を食べるのを減らしたりやめたりした時に、イライラしたり神経質になったり悲しくなったりした								
12. もし特定の食品を食べなかったことが原因で身体的な不調（例：頭痛、倦怠感等）が生じたら、不調の改善のためにその食品を食べただろう								
13. もし特定の食品を食べなかったことが原因で心の不調（例：怒り、不安、落ち込み、イライラ、落ち着かなさ等）が生じたら、不調の改善のためにその食品を食べただろう								
14. 特定の食品を食べるのを減らしたりやめたりした時に身体的な不調（例：頭痛、倦怠感等）が現れた								
15. 特定の食品を食べるのを減らしたりやめたりした時に、その食品をとても食べたくてしかたなかった								
16. 食べることに関する行動が原因で強いストレスを感じた								
17. 食品や食べることが原因で重大な問題（毎日いつも行う行動、仕事、学校、友人や家族、あるいは健康に関して）が生じた								
18. 食べ過ぎたことを後悔しすぎて他の大事なこと（仕事、家族や友人と共に過ごす等）をすることができなかった								
19. 食べ過ぎてしまったため、家族の面倒や家事ができなかった								

20. 特定の食品を食べられないという理由のため、仕事、学校、あるいは社会的な関わりを避けた								
21. 私がたくさん食べることを他の人に認めてもらえないという理由から、社交上の場面に行くのをやめた								
22. 心の不調（例：怒り、不安、落ち込み、イライラ、落ち着かなさ等）が生じても同じように食べ続けた								
23. 身体的な不調（例：頭痛、倦怠感等）が生じても同じように食べ続けた								
24. いつもと同じ量を食べた時に満足感が得られなかった								
25. 特定の食品を減らしたりやめたりしたいと本気で思ったことがあったが、できなかった								
26. いつもと同じ量を食べても良い気分が得られなかった（例：悲しさなどの良くない気分が減らなかった、満足感が得られなかった）ので、より多く食べる必要があった								
27. 食べ過ぎが原因で職場や学校で失敗した								
28. 特定の食品が身体に危険をもたらすと知っていたが、その食品を食べ続けた（例：糖尿病なのに甘い物を食べ続けた。心臓病があるのに脂肪分の多い食品を食べ続けた）								
29. 特定の食品を食べたいという欲望・衝動があまりにも強く現れて、他のことがまったく考えられなかった。								
30. 特定の食品を食べたいという欲求があまりにも強く現れて、その食品を今すぐ食べなければならないと感じた								
31. 甘い物や炭水化物等といった特定の食品群を食べることを減らしたり止めたりしようと試みたが、失敗した								
32. 特定の食品を食べることを減らしたり止めたりしようと試みた								
33. 食べることに気を取られてしまってケガをしそうになったことがあった（例：運転中、道路横断中、機械操作中に）								

34. 食品・飲料について考えることに気を取られてしまってケガをしそうになった（例：運転中、道路横断中、機械操作中に）								
35. 私の友人あるいは家族は、私がどれほど食べ過ぎるかを心配した								
